# Impact of Genetic Predisposition to Obesity on Long‐Term Maintenance of Modest Weight Loss in Postmenopausal Women

**DOI:** 10.1002/oby.70175

**Published:** 2026-03-06

**Authors:** Harold H. Lee, Christy L. Avery, Misa Graff, Daeeun Kim, Josh Arias, Linda Van Horn, Charles Kooperberg, Kari E. North

**Affiliations:** ^1^ Department of Biobehavioral Health, Pennsylvania State University University Park Pennsylvania USA; ^2^ Department of Epidemiology University of North Carolina at Chapel Hill Chapel Hill North Carolina USA; ^3^ Division of Cancer Epidemiology and Genetics National Cancer Institute Rockville Maryland USA; ^4^ Nutrition Division, Department of Preventive Medicine, Feinberg School of Medicine Northwestern University Chicago Illinois USA; ^5^ Division of Public Health Sciences Fred Hutchinson Cancer Center Seattle Washington USA

## Abstract

**Objective:**

Long‐term weight regain limits the population‐level benefits of obesity interventions. We tested whether the polygenic risk score of BMI (PRS_BMI_) modifies weight trajectories following modest weight loss.

**Methods:**

The analytic sample included 9897 postmenopausal women from the Women's Health Initiative Dietary Modification Trial (6132 European American; 3749 African American). PRS_BMI_ was derived from a trans‐ancestry GWAS of ~2 million participants. Longitudinal weight change (7 years) was modeled using weighted GEE.

**Results:**

In European Americans, the PRS_BMI_ × randomization × time interactions approached significance at the 95th percentile (*p* = 0.052) and 85th percentile (*p* = 0.07). No interaction was observed in African Americans. In analyses restricted to European Americans who lost ≥ 5% of initial weight by year 1 (20%; *n* = 1273), women in the ≥ 95th percentile of PRS_BMI_ regained nearly twice as much per year as those with average risk (0.94 vs. 0.48 kg/year, *p* = 0.0016).

**Conclusions:**

A high PRS_BMI_ was associated with faster weight regain following modest weight loss in European American women. While further validation is required in a diverse population, these results suggest the potential for genetics to inform targeted strategies for sustaining long‐term weight management.

**Trial Registration:**

ClinicalTrials.gov identifier: 75N92021D00001, 75N92021D00002, 75N92021D00003, 75N92021D00004, and 75N92021D00005

## Study Importance

- What is already known?
  ○ Initial weight loss is frequently followed by long‐term weight regain.
  ○ Recent polygenic risk scores (PRS) for obesity derived from large‐scale genetic studies, which account for approximately 5%–10% of the variation in BMI, provide increasingly actionable information for prevention.
  ○ The role of genetic risk in shaping long‐term outcomes after modest diet‐induced weight loss remains unclear.
- What does this study add?
  ○ Among 1273 European American women who reached ≥ 5% weight loss by year 1 under high‐intensity intervention (18 sessions/year), those in the top 5% of PRS_BMI_ regained 0.94 kg annually once intervention frequency decreased (4 sessions/year), which is nearly double of 0.48 kg annual regain by women at normal to low risk.
  ○ No similar association was observed among 453 African American women who achieved ≥ 5% weight loss by year 1, which suggests ancestry‐specific variability in the predictive performance of PRS_BMI_.
- How might these results change the direction of research or the focus of clinical practice?
  ○ The impact of high PRS_BMI_ on accelerated weight regain may be mediated by homeostatic counter‐regulation, which warrants further investigation.
  ○ Clinically, a PRS_BMI_ may help stratify patients for tailored weight management, including adjunct pharmacotherapy or behavioral support after initial weight loss.

## 1 Introduction

Weight regain is increasingly important, with unprecedented confidence in achieving initial weight loss through pharmacological interventions [1, 2]. While contemporary obesity interventions can produce dramatic effects in high‐risk individuals, the impacts of public policies are maximized by maintaining modest effects across the population (i.e., “prevention paradox” [3]). Identifying factors that sustain modest long‐term weight loss yields actionable insights for public policy.

The Women's Health Initiative Dietary Modification Trial (WHI‐DM) provides one such opportunity [4]. Weight regain occurs independently of the method employed for initial loss (i.e., behavioral or pharmacological) [5]. Although not designed for weight loss, in the WHI‐DM, women randomized to the low‐fat diet group experienced modest weight reductions during the first year (~2 kg on average) when intervention intensity was high (18 sessions/year), with many regaining weight over the subsequent years as intervention frequency decreased (4 sessions/year). Given that the WHI‐DM enrolled postmenopausal women, who are at increased risk of weight gain, it provides a unique opportunity to examine factors contributing to individual differences in sustained weight change following modest initial weight reduction.

The contribution of social and behavioral factors to treatment response heterogeneity is well recognized [6], but the genetic risk has been less studied. Using the latest polygenic risk scores (PRS) for obesity derived from a large genetic consortium, we examined whether genetic risk for obesity modified the effect of a low‐fat diet on long‐term weight change.

## 2 Methods

**FIGURE 1:**
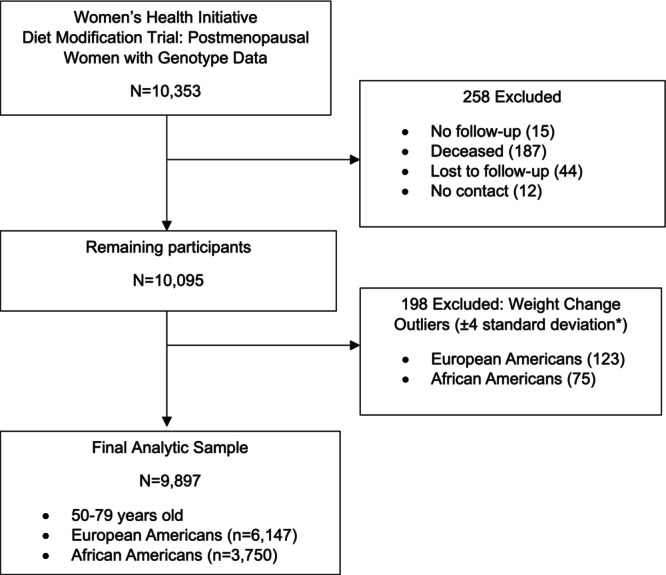
Flow diagram of study participants: European American and African American women with genotype data in the WHI Dietary Modification Trial. *Weight change outliers defined as values > 4 SD or < −4 SD from the mean.

From 48,835 participants in the WHI‐DM [4], we identified 9897 postmenopausal women aged 50–79 with genotype data: 6147 European American (63%) and 3750 African American (37%) (Figure 1). Participants were randomized to control (60%) or low‐fat diet (40%) groups. The low‐fat diet intervention emphasized maintenance of energy intake by substituting fat calories with carbohydrates. Participants were not informed of their genetic risk scores. All women provided informed consent before randomization into the trial, and the study was approved by the institutional review boards at the Fred Hutchinson Cancer Center and all 40 clinical centers.

Our outcome was visit‐specific weight difference (kg) from baseline, assessed during the 1993–2004 study period, with 7 years of median follow‐up (mean = 6.0 ± 1.8 years; range = 1–10). The polygenic risk score of body mass index (PRS_BMI_) was computed using a Bayesian continuous‐shrinkage approach [4] and an independent trans‐ancestry genome‐wide association study (GWAS) of BMI (*N* ~ 2 million; 79% European ancestry and 4% African ancestry) [7]. We operationalized PRS_BMI_ as a binary variable dichotomized at the 95th and 85th percentiles, focusing on extremes optimally with the sample size. The proportion of participants with missing weight data increased from 0.85% at baseline to 35.66% at year 7. To address potential bias from dropout, we employed weighted generalized estimating equations (GEE) to examine the impact of genetic risk on longitudinal weight change. Stabilized inverse probability weights were calculated as the ratio of two logistic regression models predicting missingness in the annual weight (kg) variable: 1 the numerator included randomization status, anthropometrics, and visit number, while 2 the denominator accounted for time‐varying predictors of missingness (diet, activity, weight change, and missingness of these covariates) along with baseline factors. To test moderation by the top 5% of PRS_BMI_ on the low‐fat diet's effect on weight change rate, weight difference was modeled using race‐specific GEE with three‐way interaction terms (PRS_BMI_ × randomization × time), adjusting for 10 ancestry‐informative genetic principal components [8], age, and baseline BMI (alpha = 0.05).

## 3 Results

At baseline, we had 6132 European Americans (age: 65.4 ± 6.6 years, BMI: 29.4 ± 6.4 kg/m^2^) and 3749 African Americans (age: 60.8 ± 6.7 years, BMI: 32.0 ± 6.6 kg/m^2^). PRS_BMI_ explained 12% of the variance in baseline BMI in European Americans and 8% in African Americans. At year 1, European Americans in the low‐fat diet group lost −2.75 ± 4.12 kg compared with −0.28 ± 3.67 kg in controls; African Americans lost −1.33 ± 4.11 kg and −0.02 ± 3.85 kg, respectively. The proportion achieving ≥ 5% weight loss was 34% in the low‐fat diet group vs. 12% in controls among European Americans and 18% vs. 9% among African Americans.

**FIGURE 2:**
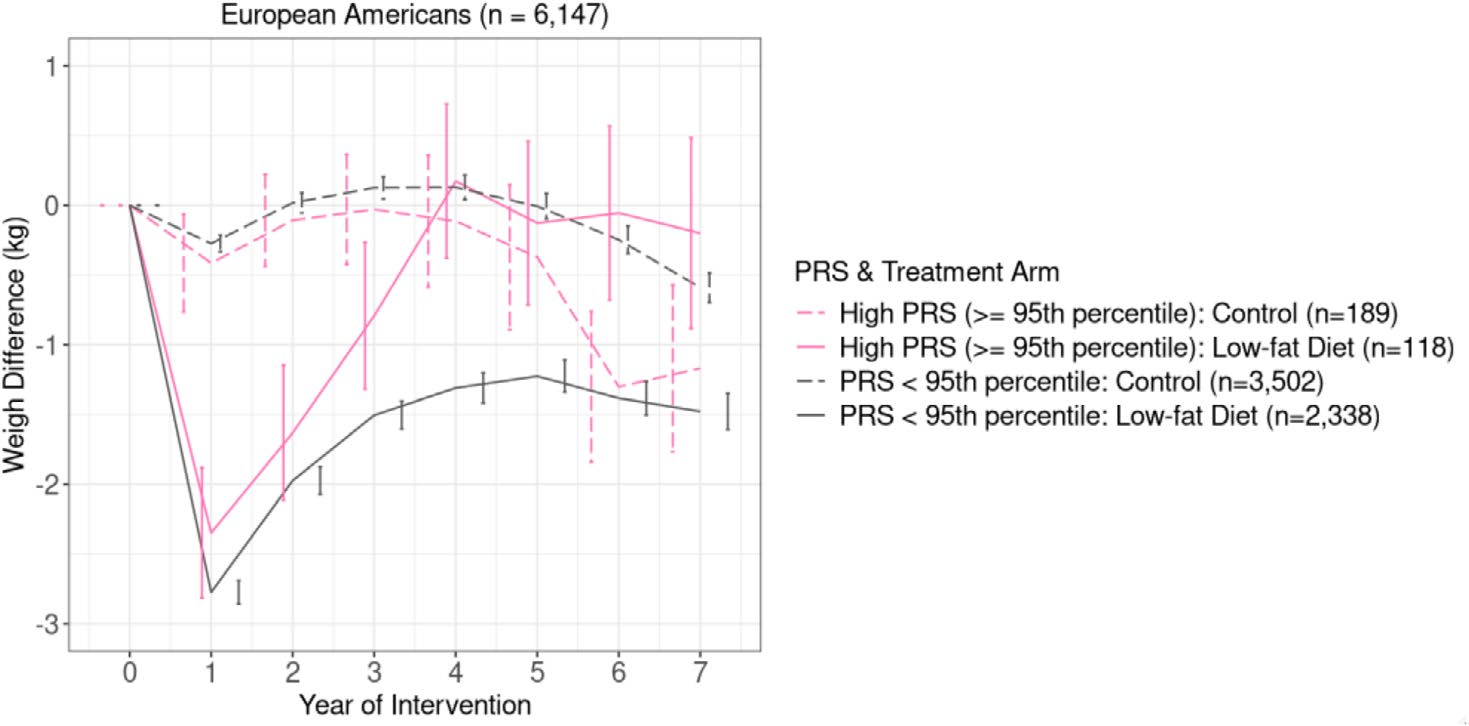
Change in body weight by intervention group and polygenic risk score (PRS) of obesity among older women of European American descent. [Color figure can be viewed at wileyonlinelibrary.com]

In European Americans, the PRS_BMI_ × randomization × time approached significance at the 95th (β = 0.32, SE = 0.16, *p* = 0.052) and 85th percentiles (β = 0.18, SE = 0.10, *p* = 0.07) of PRS_BMI_, but not with continuous PRS_BMI_. Such interaction was not observed in African Americans. Within the intervention arm, European American women at high genetic risk experienced less initial weight loss and accelerated weight regain (Figure 2). Specifically, initial weight loss from baseline to year 1 was ~0.5 kg lower than that of the remainder (*p* = 0.03), although the proportion achieving ≥ 5% weight loss was similar between those in the top 5% of PRS_BMI_ (21.4%) and the rest (22.0%). Interestingly, European American women in the top 5% of PRS_BMI_ randomized to the control group experienced more modest weight reductions over 7 years than the rest.

**FIGURE 3:**
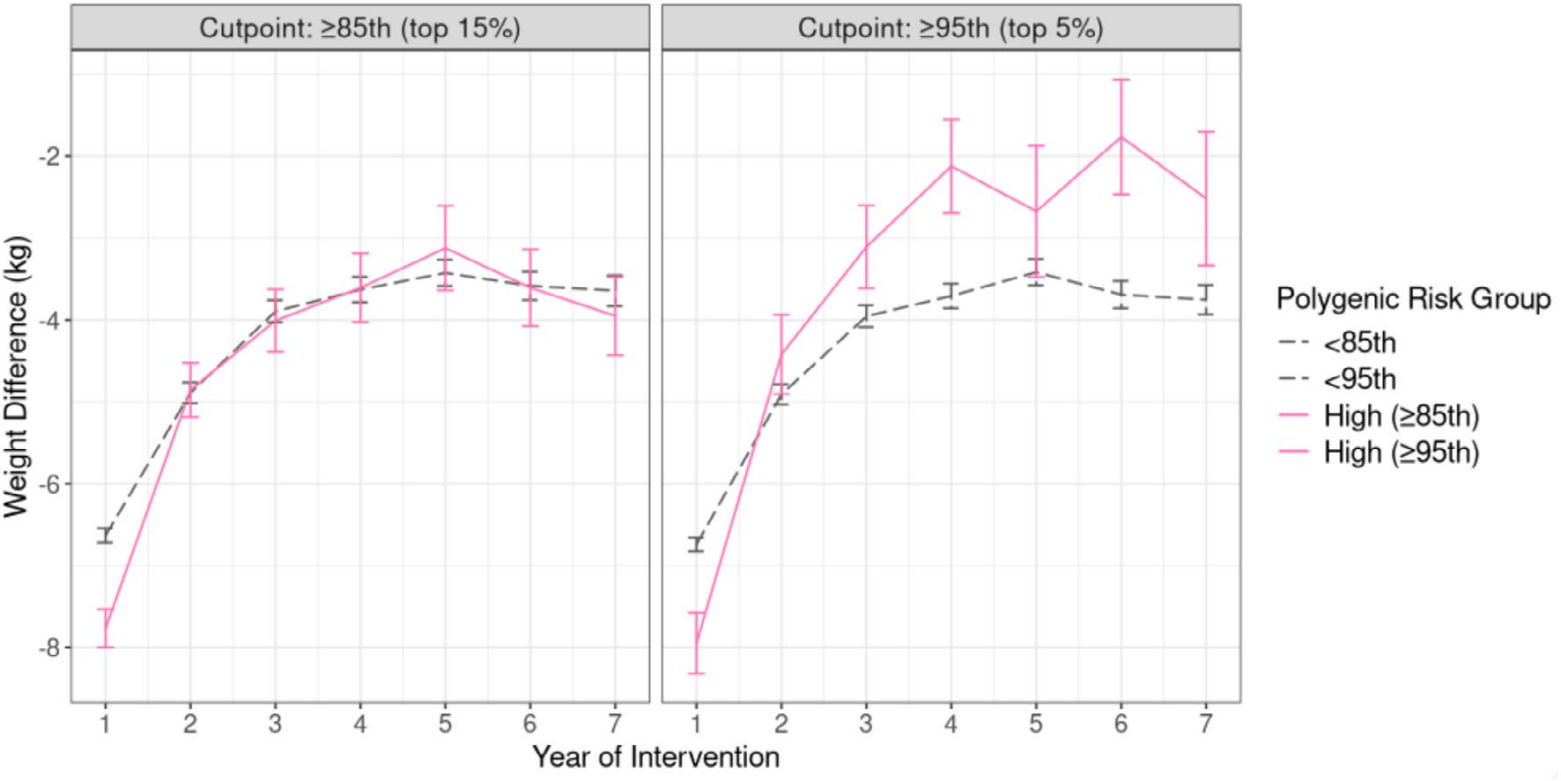
Long‐term weight regain among European Americans with ≥ 5% initial weight loss (*n* = 1273). [Color figure can be viewed at wileyonlinelibrary.com]

Importantly, WHI‐DM was not a weight loss study, and even in weight loss interventions, not everyone loses weight. To focus on weight regain exclusively among individuals who lost 5% of their initial weight, we conducted a post hoc analysis examining the genetic impact on weight regain among individuals who lost more than 5% of their initial weight. Specifically, 1273 European American participants (20%) achieved ≥ 5% weight loss by year 1, and the rate of weight regain was indeed faster among those with high genetic risk (Figure 3). That is, in the weighted GEE model additionally controlling for randomization status and initial percent weight loss from baseline to year 1, 1211 women with normal‐to‐low PRS_BMI_ (i.e., < 95th percentile) regained an average of 0.47 kg/year (*p* < 0.0001). In contrast, 62 women in the high genetic risk group (≥ 95th percentile of the PRS_BMI_) regained 0.93 kg/year (i.e., 0.45 kg/year faster; *p*‐interaction = 0.0019), nearly twice the rate observed in the normal‐to‐low PRS_BMI_ group. PRS group differences were not evident early but emerged at year 3 and persisted through year 7, with progressively greater weight regain among high‐PRS participants.

In a post hoc analysis of initial weight loss from baseline to year 1 in European American women, individuals in the top 5% of PRS_BMI_ exhibited ~0.5 kg less weight loss than the rest (*p* = 0.03). However, the proportion of participants achieving ≥ 5% initial weight loss was similar among those in the top 5% of PRS_BMI_ (21.4%) and those below this threshold (22.0%). In another post hoc analysis in European American women, we examined the genetic impact on weight regain exclusively among those who lost more than 5% of their initial weight. Because we included women in both the control and intervention arms, analyses were controlled for randomization to the intervention arm. Approximately 20% of European American participants (*n* = 1273) achieved ≥ 5% weight loss by year 1, and the rate of weight regain was indeed faster among those with high genetic risk (Figure 3). In the weighted GEE model additionally controlling for randomization and initial percent weight loss, 1211 women with PRS_BMI_ < 95th percentile regained an average of 0.47 kg/year (*p* < 0.0001). In contrast, 62 women in the top 5% of PRS_BMI_ regained 0.93 kg/year (i.e., 0.45 kg/year faster; *p*‐interaction = 0.0019). PRS group differences were not evident early but emerged since year 3 (*p* = 0.006) and persisted through year 7, with progressively greater weight regain among high‐PRS participants.

## 4 Discussion

We provide robust preliminary evidence that weight gain following a low‐fat dietary intervention varies with underlying genetic susceptibility to obesity. Our findings with faster weight regain in higher PRS_BMI_ align with a recent study in which bariatric surgery patients with higher PRS_BMI_ experienced earlier regain and less long‐term loss [9]. We observed a counterintuitive pattern in the control group, where women with high PRS_BMI_ achieved greater weight loss over the 7‐year period. For older women with high PRS_BMI_, a weak initial dose (e.g., brief educational cues) combined with long‐term nudging (e.g., repeated weight assessments) may be more effective for long‐term weight loss. Meanwhile, the slower initial loss and faster weight rebound observed in high PRS_BMI_ individuals in the intervention arm may indicate stronger homeostatic counter‐regulation to maintain baseline weight; while Figure 3 partially supports this idea, our explanation remains speculative and requires replication in long‐term trials.

The present study has limitations. First, the WHI low‐fat dietary intervention was not designed for weight loss and did not prescribe energy restriction. Our findings need replication in a trial more explicitly focused on weight loss and regain. Second, BMI may be a less precise measure of weight change in this older cohort, and some weight change may be attributable to chronic or incident diseases (e.g., diabetes, cancer) rather than the intervention itself. Finally, Hispanic participants and other racial/ethnic groups in WHI were excluded because their small numbers precluded stable estimation of the PRS × randomization × time interaction. Related, our null findings with African Americans are likely due to the smaller sample size and the reduced predictive performance of PRS_BMI_ in non‐European groups [10, 11]. Replication in more diverse populations is warranted.

PRS_BMI_ may shape both initial and long‐term responses to interventions, underscoring its utility for stratifying policies to support long‐term weight management. Future research may elucidate how people living with obesity interpret and act on genetic risk information.

## Funding

The WHI program is funded by the National Heart, Lung, and Blood Institute, National Institutes of Health, U.S. Department of Health and Human Services through 75N92021D00001, 75N92021D00002, 75N92021D00003, 75N92021D00004, and 75N92021D00005 (https://clinicaltrials.gov/study/NCT00000611). Harold H. Lee and Daeeun Kim were funded by the National Heart, Lung, and Blood Institute through T32HL129982. Support was also received from the Intramural Research Program of the Division of Cancer Epidemiology and Genetics, National Cancer Institute, National Institutes of Health. The content of this publication does not necessarily reflect the views or policies of the Department of Health and Human Services, nor does mention of trade names, commercial products, or organizations imply endorsement by the U.S. Government.

## Conflicts of Interest

The authors declare no conflicts of interest.

## Data Availability

The data that support the findings of this study are available from Women's Health Initiative. Restrictions apply to the availability of these data, which were used under license for this study. Data are available from https://www.whi.org/md/working‐with‐whi‐data with the permission of Women's Health Initiative. The statistical analysis plan for this study was submitted to the WHI Paper and Presentation Committee, which approved the plan (ms4647), and can be provided upon request.
